# Folk taxonomy and use of mushrooms in communities around Ngorongoro and Serengeti National Park, Tanzania

**DOI:** 10.1186/1746-4269-8-36

**Published:** 2012-09-21

**Authors:** Donatha Damian Tibuhwa

**Affiliations:** 1 Department of Molecular Biology and Biotechnology, University of Dar es Salaam, P.O. Box 35179, Dar es Salaam, Tanzania

**Keywords:** Ethnomycology, Kurya, Maasai, Tonic, Serengeti national park

## Abstract

**Background:**

Maasai and Kurya form two main communities around the Serengeti National Park in Tanzania which are mainly pastoralists. Changing climate to excessive drought, have recently forced them to start practicing subsistence farming which is severely affected by wild animals. This study explored status of the folk taxonomy and uses of mushrooms in the two communities as a pave way for possibilities of introducing mushroom cultivation, an alternative crop which is hardly affected by wild animals.

**Methods:**

Folk taxonomy and use mushrooms by the Kurya and Maasai communities were investigated. Information was collected by face to face interviews with 150 individuals in 6 selected villages. Using descriptive statistics by Statistic Package for the Social Science (SPSS) version 17.0, the demographic characteristics of informants were evaluated and cross relationships with the recorded data were analysed.

**Results:**

Kurya are mycophilic with 94% of the informants recognizing utilization of the wild mushroom either as foodstuff or as tonics while the Maasai are mycophobic with 99% being unaware of the edibility of mushroom although 28% recognized mushrooms as tonic. For both communities, the knowledge of mushroom utilization and folk taxonomy increased with age of the informants, while it decreases with formal education level of the informants which imply that the basis of knowledge is mainly traditional. Comparing the two communities, the Maasai use mushrooms only for medicinal purposes and never sought them for food while the Kurya were well knowledgeable on the edibility and folk classification especially the *Termitomyces* species. Characters used in folkal taxonomy included color and size of the basidiomata, shape and size of the pseudorrhiza, habitats and edibility information. A new use of ascospores whereby they anaesthaesia bees during honey harvesting was discovered, and mushroom cultivation was widely welcomed (94.7%) as an alternative crop which is rarely affected by wild animals.

**Conclusion:**

In order to salvage a noted tremendous decrease of knowledge in mushroom utilization and folk taxonomy from vanishing, there is a need to document it throughout, and incorporate it in lower levels of our education system. Mushroom cultivation may possibly be the best alternative crop for the two communities thus should be advocated for improving livelihood and reduce human wildlife conflicts. The new recorded use of ascospores to anaesthaesia the bees during honey harvesting should be exploited and scaled up for sustainable integrated bee keeping and mushroom farming.

## Background

Ethnomycology in the tropics is scarcely studied in the field of ethno biology. Perhaps because of the persistent belief that tropical mushrooms are unused and that many people in the tropics are mycophobic [1]. Folk taxonomy forms the main tool in the rare ethnomycology studies reported so far. It is the classification of organisms on the basis of cultural tradition which uses vernacular naming system. It includes knowledge such as the fruiting patterns, habitats and habits as well as uses and edibility of the organism which are vital tool for communication and information survival [2]. Folk taxonomies are generated from social knowledge and involve the way peoples make sense of, and organize their natural surroundings around them in everyday speech [2]. Folk taxonomy is distinguished from scientific taxonomy in that it is embedded within social relations and thus not objective hence un-universal.

In many parts of the word the knowledge on mushroom edibility depends much on folk taxonomy where by the knowledge is being inherited from one generation to another. In developed countries the knowledge is also obtained from taxonomic studies included in their education curriculum from their lower level of education [3]. Although common names often have a very local distribution and may change with time because of incidental events and contact with other languages, or might be too many to assign unique common names in practice [4], it remains a very useful method of communicating about mushroom usage in all local communities in Tanzania. Unfortunate in Tanzania, there is no comprehensible mushroom taxonomy syllabus in primary and secondary education [3]; this fact leaves only folk taxonomy as the popular way of classifying and communicating about mushrooms. The main tool of learning and transferring the folk taxonomy, lies on the oral traditions and is limited to the surroundings they live [5], such that when they move to a new areas the knowledge is completely lost. In Tanzania mushrooms play vital roles ecologically, socially, and economically in different communities although they are poorly studied [6]. No ethno-mycological study has been done in the country except some few uses that has been recorded on specific taxa [3,7,8].

**Figure 1:**
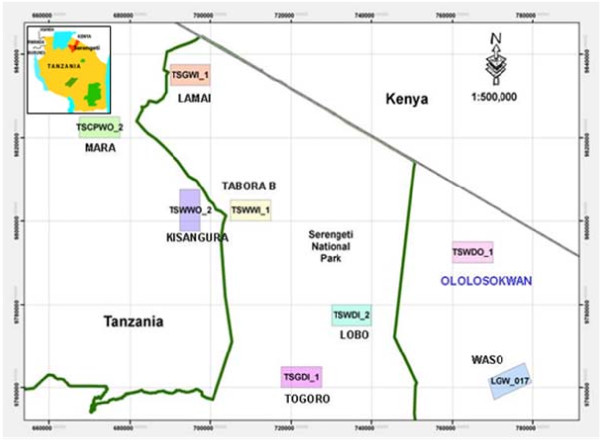
Serengeti National Park showing the study site.

The “Maasai” and “Kurya” forms the two major communities dwelling around the Serengeti National Park, Tanzania. The Kurya community lives on the western side which is wetter receiving high rainfall of c. 1200 mm per year. The Maasai live on the eastern side of the park which is relatively dry characterised by the semi-arid rangeland receiving less rainfall of c.800 mm per year (Figure 1). Maasai practice nomadic cattle herding system forming the largest group of pastoralists in East Africa, and their culture cipher precludes them eating wild animals so that their rangeland are always used by both livestock and wildlife. Because of the changing climate due to excessive drought, both communities which were previously mainly pastoralists have recently been forced to start practicing both pastoralist and subsistence farming. Since they live near the park, their subsistence farming which includes maize, cassava, millet and different legumes is severely affected by wild animals which destroy their crops. There is thus a need to explore alternative crops for the communities around the park which are hardly affected by the wild animals. *Termitomyces* mushrooms are widely distributed across the area and provide an additional source of incomes and food for the rural people especially in the western side of the park, dominated by the Kurya tribe [9]. Mushroom farming is a fast growing industry introduced in the country in early 1993 [10]. Mushrooms farming differ from other types of farming by being done in constructed huts/houses thus benefiting from not being prone to wild animals.

The present study thus aimed at: (i) explore the possibilities of introducing mushroom cultivation as an alternative crop which is hardly affected by wild animal from the park (ii) exploring dietary, therapeutic and other ethno-mycological utilizations of wild mushrooms in the studied area (iii) explore the present folk taxonomy knowledge of different communities living around the Serengeti National Park and (iv) develop a baseline data which will contribute in establishing mushroom traditional uses depository.

### Methodology

#### Study site

The study was conducted in November 2010 in two districts: Serengeti and Ngorongoro forming the North and Eastern part of the Serengeti National. The Serengeti district lies between latitude 2° 0^′^ 0^′′^S and longitude 34° 49^′^ 60^′′^E while Ngorongoro district lies between 2° 45^′^ 0^′′^S and 35° 30^′^ 0^′′^E (Figure 1). Three villages were studied in each district making a total of 6 villages for the whole study. From Serengeti studied villages included: Kisangura, Nyambuli and Machochwe, whereas in Ngorongoro the villages were Ololosokwani, Olorien Magaiduru and Soitsambu. The study area composed of highland savannah with mainly thorn woodland trees dominated by tree species in the genera *Acacia* Mill., *Comiphora* Jacq., *Ficus* L., *Combretum* Loefl. and *Podocarpus* Persoon and extensive grass plains [11]. The main tribes in the selected villages are Kurya and Maasai in Serengeti and Ngorongoro respectively although there are some immigrants from neighboring villages or other places in the country. The immigrants moved to these villages mainly because of marriage or in search of pasture, land, employments or due to relocation by the Tanzania National Parks Authority. Among the immigrant tribes found in the studied area includes the Jita, Kerewe, Haya and Nata. Crop varieties grown locally includes: maize (*Zea mays* L.), cassava (*Manihot esculanta* Crant*z*), sorghum (*Sorghum bicolor* (L.) Moench) and finger millet (*Eleusine coracana* (L.) Gaertn*)* in Serengeti, while in Ngorongoro it was mainly maize and beans (*Phaseolus vulgaris* L.). In Ngorongoro, meat and milk from their livestock is the major source of protein although bush meat is sometimes taken (Personal communication).

#### Sampling

**Figure 2:**
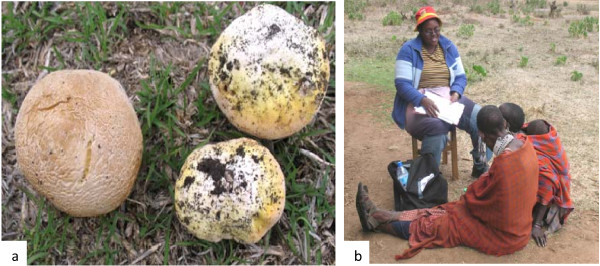
**(a) *Geastrum triplex *–used in bee harvesting (b) The author interviewing one of the participants in the left, while in the right is the translator of Swahili and Maasai languages.**

Information was collected through face-to-face interviews (Figure 2a) with 150 individuals in 6 villages belonging to 3 different wards. The work was carried out by a team of three interviewers. The sites were selected based on 2 major criteria; (i)Dominating tribes (ii) the closeness to the park where priority was given to the one more closer to the park. Information was solicited in an open-ended fashion with an inquiry form prepared by the author. Through this questionnaire, demographic features of 150 individuals were determined, some of which are described in Table 1. The interviews were generally conducted as follows: We visited village leaders and asked them to gather randomly villagers who were particularly ≥ 15 years old. In each village 5% of the house holds were interviewed. The villagers in Serengeti were mainly Kuryans while those in Ngorongoro most of them were Maasais. The questionnaires focused on: (i) the level of education (ii) main activities in the village.

**Table 1:** Demographic features of the informants n = 150

**Features**	**Frequency**	**Percentage**	**Features**	**Frequency**	**Percentage**
**Gender**			**Level of formal education**		
Male	86	57.30%	College education	3	2%
Female	64	43.70%	University education	1	0.70%
Marital status			**Employment status**		
Married	105	70%	Employed	8	5%
Single	29	19%	Farmer/pastoralist	140	94%
Widow	16	11%	Retired	2	1%
**Age**			**Tribe**		
Between 18-35	47	30%	Jita	5	3.30%
Between 36-50	88	57%	Kurya	61	40.40%
More than 50	21	13%	Sukuma	3	2%
**Level of formal education**			Masai	78	51.70%
No formal education	34	22.70%	Ikoma	1	0.70%
Primary education	97	64.70%	Kisii	1	0.70%
Adult education	5	3.30%	Haya	1	0.70%
Secondary education	10	6.70%	Pare	1	0.70%

The participants were also asked (iii) types of mushrooms they know (iv) how did they know them? (v) Edibility of mushrooms, do they use them for food? (vi) Are there any other uses they know other than food? Field guides including [3,12-14] were used wherever necessary to verify the folk taxa. The interviewer consciously avoided soliciting information on particular mushroom or particular diseases in order to prevent bias in the data collection.

#### Analysis

Results were evaluated statistically using SPSS version 17.0. Cross-relationships (i.e. how specific demographic features of the participants related to either the knowledge or the actual use of mushroom for food or tonic) were also analyzed using the SPSS statistical software.

Figure 2: (a) *Geastrum triplex* –used in bee harvesting (b) The author interviewing one of the participants in the left, while in the right is the translator of Swahili and Maasai languages.

## Results

In total, 150 individuals participated in the interview and their demographic features are summarized in Table 1. There were 43.7% females and 57.3% males and all the participants had lived in the area for more than 5 years. In Serengeti, 71 individuals (males: 61%, n = 43, females: 39%, n = 28) whereas in Ngorongoro 79 individuals were interviewed (males: 54%, n = 43, females: 46%, n = 36).

**Figure 3:**
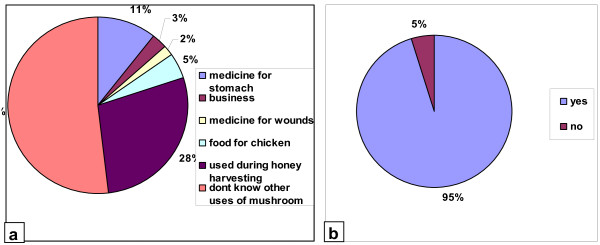
(a) Other uses of mushroom apart from food (b) Willingness to participate in mushroom cultivation as alternative crop rarely affected by wild animals.

The respondents’ ages ranged from 15 to 87 years. The Kurya was the dominant tribe in Serengeti (86%, n = 61), among the respondents while the rest were Jita, Sukuma, Ikoma, Kisii or Haya, Kerewe, and Nata. In Ngorongoro the Maasai tribe was dominant (99%, n = 78) and only one person was found to be a Pare. In terms of educational level, most of the interviewed people had primary education (Serengeti: 86%, n = 61; Ngorongoro: 46%, n = 36). There were a number of people who had no formal education in Ngorongoro with about 39% (n = 31) of the interviewees, whereas in Serengeti the percentage was low i.e. 4%) (n = 3). The other 10% in Serengeti and 15% in Ngorongoro had secondary, adult, college or university education. In the Serengeti district wild mushrooms were found mainly used for food or to alleviate or to treat symptoms or diseases such as abdominal pains, wounds, poor health due to long illness and breast feeding mothers. The information on the dietary, therapeutic, and other ethnomycological uses of mushrooms are summarized in Table 2 and Figure 3.

**Table 2:** Indigenous knowledge on mushroom utilization and folk taxonomy

**Family**	**Species**	**Ethnotaxa**		**Traditional uses and preparations**		
		**Kurya**	**Masai**	**Masai**	**Kurya**	**Local Preparation and administration**
Lyophyllaceae	*Termitomyces microcarpus* (Berk & Broome) R.Heim	Bitoghose	Not known	Not known	Food, improve healthy to long ill people and breast feeding mothers	Cooked with spices as onion cooking oil and tomatoes and milk
Lyophyllaceae	*T. titanicus* Pegler & Piearce	Lyugu	Ormambuli	Few know it as tonic various gastro- intestinal ailments (e.g., abdominal pain, constipation, stomach ache and ulcers),	Food, delicacy for important members of the community; tonic various gastro-intestinal ailments	Boiled and added salt to taste
Lyophyllaceae	*T. aurantiacus* (R. Heim) R. Heim	Nyankobhiti	Ormambuli	Not known	Food, tonic for stomach aching	Boiled and added salt to taste
Lyophyllaceae	*T. clypeatus* R*.* Heim	Vihungumururyo	Ormambuli	Not known	Food	Cooked with spices such as onion cooking oil and tomatoes
Lyophyllaceae	*T. eurhizus (Berk)* R. Heim	Amanyegiswa	Ormambuli	Not known	Food	
Lyophyllaceae	*T. le-testui* (Pat.) R. Heim	Lyugu	Ormambuli	Few know it as tonic various gastro- intestinal ailments (e.g.,abdominal pain, constipation, stomach ache and ulcers),	Food	Cooked with spices such as onion cooking oil and tomatoes
Lyophyllaceae	*T. mammiformis* R. Heim	Bitoghose	Ormambuli	Not known	Food	
Lyophyllaceae	*T. umkowaan* (Cooke & Massee) D.A Reid	Amughu	Ormambuli	Few know it as tonic various gastro- intestinal ailments (e.g.,abdominal pain, constipation, stomach ache and ulcers),	Food	Cooked with spices such as onion cooking oil and tomatoes
Lyophyllaceae	*T. tylerianus* Otieno	Bitoghose	Ormambuli	Not known	Food	Cooked with spices such as onion cooking oil and tomatoes
Lyophyllaceae	*T. striatus (Beeli)* R. Heim	Bitoghose	Ormambuli	Not known	Food	Cooked with spices such as onion cooking oil and tomatoes
Agaricaceae	*Agaricus campestris* L.:Fr.	Bitoghose	Ormambuli	Not known	Food	Cooked with spices such as onion cooking oil and tomatoes
Agaricaceae	*A. bisporus*	Nyankobhiti	Ormambuli	Not known		
Agaricaceae	*A. xanthodermus*	Nyankobhiti	Ormambuli	Not known	Not known	None
Agaricaceae	*C. comatus* (O.F. Müll.) Pers	Nyankobhiti	Ormambuli	Not known	Not known	None
Agaricaceae	*Cholorophyllum*	Binyankorogoto	Ormambuli	Not known	Not known	None
	*molybdites* (G. May.)	Binyankorogoto	Ormambuli	Not known	Not known	None
Agaricaceae	*P. tuber-regium (*Rumph. Ex Fr.)	Binyankorogoto	Ormambuli	Not known	Not known	None
	Singer					
Agaricaceae	*Macrolepiota procera* (Scoop.) Singer	Binyankorogoto	Not known	Not known	Healing wounds	The bulbolous base is dried and grinded to powder form which is then applied direct to the wound.
Agaricaceae	*Trametes versicolor* (L.)Lloyd	Binyankorogoto	Ormambuli	Not known	Not known	None
Polyporaceae	*Trametes elegans*	Binyankorogoto	Not known	Not known	Not known	None
Polyporaceae	(Spreng.)Fr.	Binyankorogoto	Not known	Not known	Not known	None
Schizophyllacea	*Schizophyllum commune* Fr.	Binyankorogoto	Not known	Not known	Not known	None
Ganodermataceae	*Ganoderma boninense* Pat.	Binyankorogoto	Not known	Not known	Treat wound and skin infection	Use its powder to treat skin infection and wound
Geastraceae	*Geastrum saccatum* sensu auct. Brit.	Uiborinyiti	Not known	Subject the bees into ‘anaesthaesia’ state	Subject the bees into ‘anaesthaesia’ state	Pierce the ascocarp (ball) to release spores into the bee hives
Geastraceae	*G. triplex* Jungh.	Uiborinyiti	Not known	Subject the bees into ‘anaesthaesia’ state	Subject the bees into ‘anaesthaesia’ state	Pierce the ascocarp (ball) to release spores into the bee hives

The mushroom species are listed, in the first section, in alphabetical order by genus name corresponding to the indigenous name from different tribes. Seven uses of 12 mushrooms belonging to 4 genera were recorded. Five species were used for therapeutic purposes *T. microcarpus*, *T*. *letestui*, *T. titanicus, T. mammiformis*, *P. tuber-regium* while two *Ascomycetes* (*G. triplex* and *G. saccutum*) contained most of the utilized species.

The Eastern part of the Park which is mainly occupied by the Maasai community traditionally never eats mushrooms by their culture. However in this study, 28% of the participants reported that they use *Ascomycetes* spores referring them to as ‘soots’ to *anaesthaesia* the bees during honey harvesting, and 75% of this group reported that they do so frequently. Only 3 people who actually lived out of their area confirmed to know the edibility of mushrooms.

## Discussion

### Mushrooms uses

As shown in Table 2 the Kurya tribe recognized wild mushrooms uses as either foodstuff or as tonic, with only few who were unaware of that. Among the mushrooms used for therapeutical purposes, 4 mushrooms species (*T. titanicus, T. letestui, T. eurhizus, T. aurantiacus*) were used to treat various gastro-intestinal ailments (e.g., abdominal pain, constipation, stomach ache and ulcers). *Termitomyces microcarpus* was found to be used in immune boosting. It is given to sick people for quick recovery and lactating mothers. Utilizations stated in this survey were compared with already known uses of mushrooms such as those stated in [4,15-20]. A new use of mushroom was established; the use of *Ascomycetes* spore (soot) in honey harvesting. The ascospores which when mature dry and burst out realizing the ascospores which comes out like soot, is directly opened into the beehives. The “soot” in contact with the bees make them unconscious like subjecting them under ‘*anaesthaesia’* for a about 30 minutes allowing the honey harvester to complete their task safely. This technology can be widely adopted for sustainable honey harvesting as it does not harm the bees. Results from this study also show that 95% of participants welcome the idea of mushroom cultivation which is hardly affected by wild animals (Figure 3). Interestingly, apart from the monkeys, baboons and tortoise which were found eating mushrooms, other wild animals seemed not to eat them, as they were found almost undisturbed inside the park in vicinity of troops of wild animals such as elephants, wilder beast, Tommy gazelle and buffalos. This will probably help reducing human wildlife conflict while improving living standard of the people around the park.

### Mycophily and mycophoby

From the total of interviewees, Kurya community from Serengeti (80% of informants), possessed a more detailed local mycological knowledge as well as a wider acceptance as an edible resource. They provided a lot of information when asked about mushrooms edibility, folk taxonomy and any other ethnomycological uses (Table 2, Figure 3). On the other hand, Maasai tribe from Ngorongoro (72% of informants) did not give much information; in fact they showed certain mistrust talking about the topic, showing no interest, even dislike and even expressed their phobia to the mushrooms. This observation is in contrast with the observations from studies by Mapes et al. [21] in Amazonia and Mesoamerica and Goes-Neto and Bandeira in Brazil [22] who established that mycophoby is palaetropical based. In this study a widespread mycophoby or non-mycophily among inhabitants of the studied area (Maasai) is rather a differential empathy related to ethnic origin and not palaeotropical based. Maasai informants from Ngorongoro can be classified as mycophobic while Kurya informants from Serengeti can be classified as non-mycophobic. Although some of the interviewees were not mycophilic, a percentage of them were truly mycophilic and they all shared the fact of being indigenous and inhabitants of the studied area. Although different authors claim the presence of a clear difference among inhabitants of temperate areas and tropical areas concerning their traditional mycological knowledge and practices (21); this study found two groups mycophobic and mycophilic based on their ethnic groups within the tropical areas.

### Folk and scientific classification

The Kurya tribe whom this study found mycophilic, their natural folk taxonomies especially species of the genus *Termitomyces* were identical to some level, especially the species, with those recognized by western sciences. This result is in line with the finding by Bulmer and Tyler [23-25], who also noted similar trend of ethnotaxa being comparable to scientific taxa. The characters which were found used in distinguishing the ethnotaxa were color and size of the basidiomata, shape and size of the pseudorrhiza, habitat such as termite mounds associations and leaf litter, as well as the edibility information.

It should be noted that the taxa which occur as members of the specific and variety of the ethnobiological categories differ from both life form and generic taxa in several respects. Many folk taxa are conceptually distinguished on the basis of very few morphological characters. For example, the *Termitomyces* species with folk taxa *T. titanicus*- ‘lyugu’, *T. microcarpus*- ‘bhitoghose’ *T. clypeatus*- ‘vihungumururyo’; *T. eurhizuz*- ‘amanyegiswa’ *T. aurantiacus* ‘nyankobhiti’and any other mushroom which is not thought edible ‘binyankoroghoto’ for Kurya while the Jita and Kerewe tribe call them ‘matebe ga nyamikolo’ meaning wild bird’s chair. The Jita and Kerewe although they were minority tribes in this study, they showed a great fear to the mushroom, and in-fact they surely explained that they never eat mushrooms the belief which is translated by the name that mushrooms are wild bird’s chair. This mockery altitude may also be associated by their ethnic originality as they come from surrounding Lake Victoria, the second biggest lake in the world, thus enjoy free fish harvesting which is more delicious compared to mushrooms.

The ethnotaxa observed in this study were polytypic with the same term used to refer to more than one species (Table 2). This observation concur with that of Berlin (24, 2), who named it as ‘under- differentiation’ and corresponded it with the western scientific classification. He also noted that the under-differentiation is more practiced among traditional societies that manage their resources mostly for food.

The result of the folk taxonomy knowledge shows a tremendous decrease with age. Modernization which includes taking children to boarding schools thus keeping them away from their elders most of the time also play a key factor which contributes to the loss of folk taxonomy knowledge. There is still much to be learned about ethnomycological uses of mushroom from local and indigenous peoples who have, for many generations, managed and used mushroom resources especially in areas of high biodiversity and in developing mitigation strategies to cope with changing climate. For a variety of reasons, much of this knowledge is being lost and that is something that should not be allowed to happen. The knowledge should rather be recognized and people who have it need to be brought into the conversation and respected for what they know. The scientists should cooperate by providing their assistance where applicable in order to salvage this information and knowledge from peter out.

## Conclusion

Based on the data presented in this study, it appears that the Kurya community is mycophilic while the Maasai is mycophobic. From the result analysis this phobic is rather sympathy of ethnic origin and culture based but can be easily mitigated with education on the use and importance’s of mushrooms. In order to reduce wildlife human conflicts and improve the living standard of the people around park by the use of subsistence agriculture, introduction of mushroom farming which is rarely affected by wild animals is essential. From the study results showed decreasing folk taxonomy knowledge with decreasing age, it is important to include these lessons in the lower level of the educational system for the purposes of not loosing this valuable knowledge which is the only basis of taxonomy in most country side. The new recorded use of ascospore in bee harvesting should be exploited and scaled up for mass productions of sustainable integrated bee keeping and mushroom farming.

## Competing interests

The author declare that he has no competing interests.
